# Supplementary figures and images for: Bacillus subtilis-Derived Postbiotics as a Multifunctional Bio-Catalyst for Enhancing Lactic Acid Bacteria Viability and Yogurt Quality
Source: Foods. 2025 May 19;14(10):1806. doi: 10.3390/foods14101806 (PMC12111040; doi:10.3390/foods14101806)

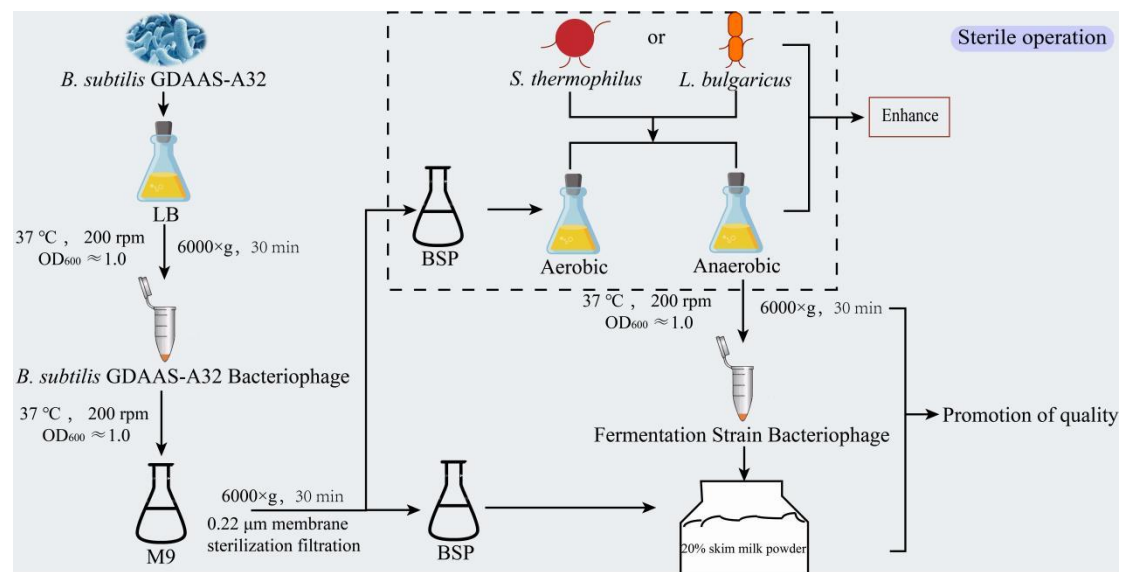

**Figure S1.** BSP preparation and yogurt production workflow.

Supplement: Supplementary file 1 [file foods-14-01806-s001.zip › foods-3631662-supplementary.pdf]
